# Brown Adipose Tissue Bioenergetics: A New Methodological Approach

**DOI:** 10.1002/advs.201600274

**Published:** 2017-03-13

**Authors:** María Calderon‐Dominguez, Martín Alcalá, David Sebastián, Antonio Zorzano, Marta Viana, Dolors Serra, Laura Herrero

**Affiliations:** ^1^ Department of Biochemistry and PhysiologyInstitut de Biomedicina de la Universitat de Barcelona (IBUB)Universitat de BarcelonaE‐08028BarcelonaSpain; ^2^ Centro de Investigación Biomédica en Red de Fisiopatología de la Obesidad y la Nutrición (CIBEROBN)Instituto de Salud Carlos IIIE‐28029MadridSpain; ^3^ Facultad de FarmaciaUniversidad CEU San PabloE‐28668MadridSpain; ^4^ Institute for Research in Biomedicine (IRB Barcelona)The Barcelona Institute of Science and TechnologyDepartament de Bioquímica i Biomedicina MolecularFacultat de BiologiaUniversitat de BarcelonaE‐08028BarcelonaSpain; ^5^ Centro de Investigación Biomédica en Red de Diabetes y Enfermedades Metabólicas Asociadas (CIBERDEM)Instituto de Salud Carlos IIIE‐28029MadridSpain

**Keywords:** bioenergetics, brown adipose tissue, mitochondria, oxygen consumption rate, seahorse

## Abstract

The rediscovery of brown adipose tissue (BAT) in humans and its capacity to oxidize fat and dissipate energy as heat has put the spotlight on its potential as a therapeutic target in the treatment of several metabolic conditions including obesity and diabetes. To date the measurement of bioenergetics parameters has required the use of cultured cells or extracted mitochondria with the corresponding loss of information in the tissue context. Herein, we present a method to quantify mitochondrial bioenergetics directly in BAT. Based on XF Seahorse Technology, we assessed the appropriate weight of the explants, the exact concentration of each inhibitor in the reaction, and the specific incubation time to optimize bioenergetics measurements. Our results show that BAT basal oxygen consumption is mostly due to proton leak. In addition, BAT presents higher basal oxygen consumption than white adipose tissue and a positive response to b‐adrenergic stimulation. Considering the whole tissue and not just subcellular populations is a direct approach that provides a realistic view of physiological respiration. In addition, it can be adapted to analyze the effect of potential activators of thermogenesis, or to assess the use of fatty acids or glucose as a source of energy.

## 1 Introduction

Interest in the study of brown adipose tissue (BAT) has been reawakened in the past few years. Traditionally, the presence of this type of adipose tissue was considered residual in adult humans, and it was thought to be almost exclusively present in neonates. However, in 2009, several independent research groups used positron emission tomography‐computed tomography scans to identify not only the presence of BAT in adult humans, but also the possibility of inducing its metabolic activity.^1^, ^2^, ^3^, ^4^, ^5^, ^6^, ^7^ Consequently, increasing efforts have been dedicated to researching BAT as a possible therapeutic target in the treatment of metabolic diseases such as obesity or diabetes.^8^, ^9^

Like white adipose tissue (WAT), BAT can store triglycerides, although its main function is to oxidize them and dissipate the resulting energy as heat in a process called nonshivering thermogenesis. The biochemical reason for this activity is the high number of densely packed mitochondria in the brown adipocytes, together with the high expression of uncoupling protein 1 (UCP‐1). Upon activation, UCP‐1 acts as a proton channel within the inner mitochondrial membrane, pumping back protons from the intermembrane space toward the mitochondrial matrix. This avoids adenosine triphosphate (ATP) synthase and thus dissipates the energy generated as heat.

The sympathetic nervous system is the main activator of BAT. When norepinephrine binds to the β3‐adrenergic receptor, the levels of cyclic adenosine monophosphate (cAMP) increase, activating protein kinase A (PKA). This leads to increased lipolysis and the resulting fatty acids, which provide the fuel for mitochondrial β‐oxidation^10^ and are allosteric activators of UCP‐1.^11^ Furthermore, PKA promotes the induction of UCP‐1 expression through phosphorylation of p38 MAPK (mitogen‐activated protein kinase) and cAMP‐response‐element‐binding.^12^, ^13^

In addition to sympathetic control, some novel BAT activators have been identified recently.^9^, ^14^ These include activators of nuclear hormone receptors, such as vitamin A‐related molecules or peroxisome proliferator‐activated receptor (PPAR) inducers.^15^, ^16^ Other endocrine factors, such as cardiac natriuretic peptides,^17^ irisin released from skeletal muscle,^18^ or hepatic fibroblast growth factor‐21,^19^ have also been proposed to work as BAT activators.

Due to increased interest in the activation of this tissue, novel techniques to study BAT bioenergetics are required. The consumption of O_2_ in the mitochondria can be measured by several methods, including Clark‐type oxygen electrodes,^20^ the Oroboros Oxygraph‐2k,^21^ and fiber optic oxygen sensors.^22^ However, the XF24 Extracellular Flux Analyzer (XF Seahorse Bioscience, North Billerica, MA) has several advantages. It can be used with small sample sizes, simultaneously detects the oxygen consumption rate (OCR) and the extracellular acidification rate (ECAR) in 24‐well or 96‐well plates, and allows the use of replicates and multiple conditions. In addition, it avoids the mitochondrial rupture during the isolation process, one of the main disadvantages in the mitochondrial isolation protocols. Although the XF24 Extracellular Flux Analyzer method has been commonly used in isolated mitochondria and cultured cells,^23^, ^24^ its application in tissue explants would provide deeper, clearer insight into physiological respiration.

In the present study, we show how to adapt the protocol using the XF24 Islet Capture Microplate in fresh explants of interscapular BAT (iBAT) from C57BL6/J mice, a common rodent model used in metabolic studies. Parameters such as the amount of tissue, the dosage of the inhibitors, and incubation times were established to obtain more accurate results. This approach, which yields bioenergetic information about basal respiration, ATP turnover, proton leak, maximal respiration, or spare respiratory capacity, provides a more accurate physiological overview of the respiratory status within the tissue than previous models using cultured cells or isolated mitochondria.

## 2 Results and Discussion

### 2.1 Ex Vivo Measurement of Cellular Bioenergetics Profile in Brown Adipose Tissue Explants

Several experiments were performed to titrate the key parameters and thus determine OCR in iBAT explants. Variables such as the number and weight of the explants, the concentration of each inhibitor, and the reaction and incubation times were tested. Cutting the tissue in several pieces would provide an increased surface, improving the absorption of the inhibitors. However, the best results were achieved using 9 mg of tissue in one single punch, since lower amounts often resulted in floating tissue inside the wells, leading to incorrect readings. We also established the optimal concentration of the inhibitors (24 µg mL^−1^ oligomycin, 0.8 × 10^−6^ m carbonyl cyanide‐4‐(trifluoromethoxy)phenylhydrazone (FCCP), 5 × 10^−6^ m rotenone, 15 × 10^−6^ m antimycin A) and the stabilizing and injection times, which are described in **Table** **1**.

**Table 1:** Seahorse XF24 run protocol

Command	Number of loops/cycles	XF24 times [min]
Calibrate	–	–
Equilibrate	Auto	Auto
Mix, wait, measure	5	3, 2, 3
Inject A	–	–
Mix, wait, measure	4	3, 2, 3
Inject B	–	–
Mix, wait, measure	8	3, 2, 3
Inject C	–	–
Mix, wait, measure	5	3, 2, 3

**Figure 1:**
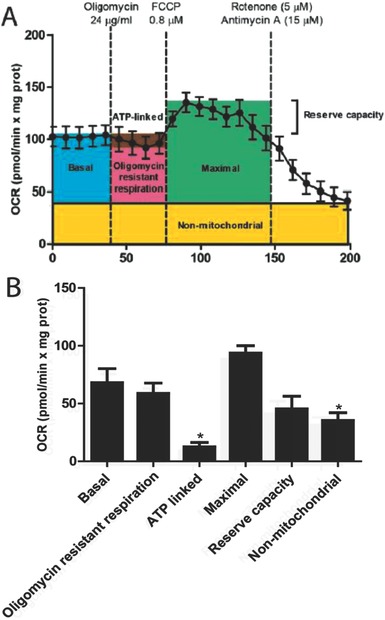
Oxygen consumption rate in brown adipose tissue explants. 9 mg of iBAT from 13 mice were analyzed using the XF24 Islet Capture Microplate. The amount of tissue, concentration of inhibitors, and incubation times were used as described in the Experimental Section. The injection of inhibitors is represented as dashed lines. A) Average OCR trace, depicting basal oxygen consumption, oligomycin resistant respiration (H^+^ leak), and ATP‐linked OCR after the addition of 24 µg mL^−1^ oligomycin. Maximal respiratory rate and reserve capacity were calculated after the injection of 0.8 × 10^−6^ m FCCP. Finally, 5 × 10^−6^ m rotenone and 15 × 10^−6^ m antimycin A were added to observe nonmitochondrial respiration. B) Bioenergetic parameters were inferred from the OCR traces. The results are represented as mean ± standard error of the mean (SEM). * Shows statistical differences between bioenergetic parameters and basal respiration. *N* = 13, *p* < 0.05.

Once the conditions had been established, we measured the OCR in iBAT from 13 C57BL6/J mice. The results are represented in **Figure** **1**A, which shows the trace of the OCR over time and the effects of the different inhibitors. We first incubated the tissue in assay media for 45 min prior to the analysis of the OCR. Once the OCR had stabilized, we programed five readings to record the basal respiration. Then, oligomycin (an ATP synthase inhibitor) was injected, to distinguish between respiration dedicated to the synthesis of ATP (referred to as ATP‐linked respiration) and non‐ATP linked consumption of oxygen. Typically, OCR decreases after oligomycin injection, depending on the degree of oxygen consumption used for ATP synthesis. It also induces respiratory state 4, causing an increase in the mitochondrial membrane potential (Δψ_m_) and contributing to increased proton leak through the membrane.^25^, ^26^ This may result in overestimation of the non‐ATP linked OCR, but may only have a brief impact in the case of iBAT, which is already rich in uncoupling channels.

After FCCP addition, oxygen consumption is stimulated to reach the maximal OCR. With the addition of this uncoupling agent, the electron transport chain (ETC) is no longer controlled by the proton gradient across the mitochondrial inner membrane. Finally, a mix of rotenone and antimycin A, both inhibitors of complexes in the electron transport chain, were added. Blocking the transport of electrons in the ETC leads to complete depletion of oxygen consumption in this chain, so the remaining oxygen consumption is preferentially due to extramitochondrial oxygenases or reactive oxygen species generation.

The raw data of the OCR obtained in this experiment are represented in Figure 1B. Our data have similar magnitudes to previous reports using the XF Seahorse technology in cultured cells^27^ or the Clark‐type oxygen probe in BAT isolated mitochondria after tissue weight normalization.^28^ Compared to previous observations in WAT,^29^ iBAT shows a similar OCR corresponding to nonmitochondrial respiration, related mainly to the action of oxygenases.^30^ Regarding mitochondrial respiration, non‐ATP‐linked respiration was sevenfold higher than ATP‐linked respiration, which reflects the high content of uncoupling proteins within the mitochondria of iBAT.

### 2.2 Cellular Respiratory State Calculations Confirm Electron Transport Chain Uncoupling in Brown Adipose Tissue Explants

The cellular respiratory state in most cell mitochondria varies from state 3 (when both adenosine diphosphate (ADP)) and substrate levels are high, and consequently the respiration rate is also increased) and state 4 (when the respiration rate decreases due to low levels of ADP in spite of high levels of substrate). This transition is modulated by changes in basal OCR, the coupling status, the maximal respiratory rate, or changes in the activation or levels of expression of proteins controlling respiration, among other factors. To calculate the apparent respiratory state, we used the Equation (1)

(1) $${\mathrm{State}}_{\mathrm{apparent}}=\;4\;\;\frac{\left(\mathrm{Basal}\;\;\mathrm{Oligo}\right)}{\left(\mathrm{FCCP}\;\;\mathrm{Oligo}\right)}$$

Where Basal represents the basal OCR, Oligo represents the OCR after oligomycin addition, and FCCP represents the FCCP‐stimulated OCR.^31^ According to this formula, the apparent cell state in our model was 3.44, which suggest an intermediate respiration rate and ATP synthesis at the time of sacrifice.

In addition to the cellular respiratory state, the respiratory control rate (RCR), defined as the extent to which respiration is slowed down once the ADP has been completely phosphorylated, was defined as a gold standard for mitochondrial coupling. The bioenergetics profile can be used to calculate the RCR for oxidative phosphorylation by assuming that respiratory states 3 and 4 are achieved after oligomycin and FCCP, respectively^31^

(2) $${\mathrm{RCR}}_{\mathrm{basal}}=\;\frac{\left(\mathrm{Basal}\;\;\mathrm{Anti}\;A\right)}{\left(\mathrm{Oligo}\;\;\mathrm{Anti}\;A\right)}$$

(3) $${\mathrm{RCR}}_{\max}=\;\frac{\left(\mathrm{FCCP}\;\;\mathrm{Anti}\;A\right)}{\left(\mathrm{Oligo}\;\;\mathrm{Anti}\;A\right)}$$

In our model, RCR_basal_ (Equation (2)) and RCR_max_ (Equation (3)) were 1.25 and 1.45, respectively. This indicates that the ETC and oxidative phosphorylation are highly uncoupled in our model, which is a typical characteristic of iBAT.

It is important to note that some of the potential applications of this method may require further normalization of the bioenergetic data beyond controlling the tissue weight, as several metabolic conditions may involve different degrees of fat deposition within the tissue. Here, we represent our results normalized by protein content, but other parameters, such as relative mitochondrial DNA copy number may be used to normalize data.

### 2.3 Potential Applications of Bioenergetics Measurement in Brown Adipose Tissue

**Figure 2:**
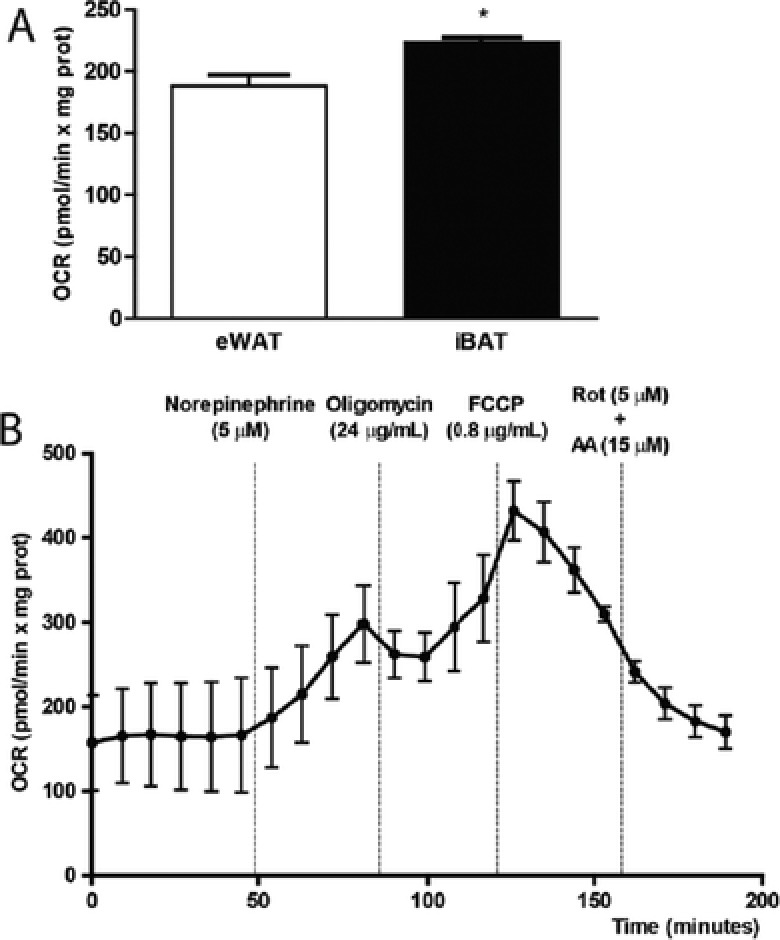
Potential applications of bioenergetics measurement in brown adipose tissue. A) Comparative analysis of basal oxygen consumption in epididymal white adipose tissue (eWAT) and interscapular brown adipose tissue (iBAT). Basal OCR was analyzed in equal amounts of eWAT and iBAT, using the XF24 Islet Capture Microplate and normalized by milligrams of protein. B) OCR trace in iBAT explants after norepinephrine injection. The injection time and the concentrations used for the different compounds are shown. Rot: Rotenone. AA: Antimycin A. Results are represented as mean ± SEM. * Shows statistical differences between eWAT and iBAT. *N* = 13, *p* < 0.05.

As an example of the potential applications of this method, we compared the basal OCR of iBAT from ten mice with their corresponding epididymal WAT (eWAT) (**Figure** **2**A). Basal OCR in iBAT was increased compared to eWAT. This could be explained by increased ATP turnover within the cells, by natural differences in the proton leak because of the high expression of UCP‐1 in iBAT, or by an increase in nonmitochondrial reactive oxygen species (ROS) production.^31^ In addition, we tested our method to evaluate the iBAT respiration response to β‐adrenergic stimulation. Norepinephrine was able to induce a twofold increase in BAT respiration (Figure 2B). This supports the application of our method to detect changes in respiration following BAT stimulation.

Future applications of this method may include the comparison of bioenergetics between different metabolic states, treatment effects, or genetic models. The method can also be modified to investigate the impact of several effectors on iBAT activation by adding pretreatment steps to the tissue explants. Additionally, the fuel consumption of iBAT can be determined by coupling this detection method with the use of palmitate as a substrate. This allows the quantification of both endogenous and exogenous fatty acid oxidation. Finally, since glucose is also an important source of energy for this tissue,^32^ the method can be adapted to determine glycolysis in real time, by detecting the ECAR.

## 3 Conclusion

We believe that this method improves bioenergetics measurement for the following reasons: (1) the use of a whole tissue explant better reflects the physiological situation and avoids the mitochondrial isolation steps that commonly lead to mitochondrial rupture; (2) the use of a single punch of tissue avoids the misplacement of tissue in the plate, and thus leads to more accurate readings; (3) the concentration of the inhibitors is adapted to the specific characteristics of iBAT and does not require previous permeabilization of the tissue; (4) a wide range of information can be retrieved by using simple adaptations of this method (for example, bioenergetic parameters can be compared, and the influence of effectors or inhibitors on fuel utilization can be assessed).

This method can also be adapted for other tissues in animal or human models. Nonetheless, we recommend testing the proper normalizing parameter (weight of tissue, protein content, mitochondrial DNA) that best fits each specific situation.

## 4 Experimental Section

*Preparation of the Sample*: Eight‐week‐old male C57BL6/J mice were sacrificed by cervical dislocation after overnight fasting. All the procedures were performed in accordance with the University of Barcelona's Ethical Committee for Animal Research.

iBAT and eWAT were excised and placed in small petri dishes containing wash media (eagle's minimal essential medium (DMEM) (L0102‐500, Biowest) supplemented with 25 × 10^−3^ m glucose and 25 × 10^−3^ m 4‐(2‐hydroxyethyl)‐1‐piperazinemethanesulfonic acid (HEPES)). Hair and blood were removed by gentle agitation, and tissues were rerinsed in fresh wash media. 9 mg tissue samples were acquired using a scalpel from the same section of tissue. Tissues were kept in wash media until the respiration assay.

*Application of Tissue to Assay Plate*: The capture screens (Seahorse Bioscience, North Billerica, MA) were prewet in wash media in a small petri dish to remove any air bubbles. A pair of sterile forceps was used to position the screens (ring facing up), and a total of 9 mg of iBAT was placed on the screen with forceps. Then, the capture screen insert tool (Seahorse Bioscience, North Billerica, MA) was used to pick up the tissue‐containing screens from the petri dish, and to place them in an XF24 Islet Capture Microplate (Seahorse Bioscience, North Billerica, MA). Once in position, all the tissues samples were rinsed twice with wash media, and once with assay media (AM, DMEM with 25 × 10^−3^ m glucose). Finally, 450 µL AM was added to all the samples and control wells.

*Oxygen Consumption Rate Measurement*: The Seahorse XF24 (Seahorse Bioscience, North Billerica, MA) was used to measure the OCR in real time. This system measures changes in oxygen concentration in a small amount of media that is isolated above the tissue. For a typical bioenergetic profile, a set of inhibitors (Sigma‐Aldrich) of key components of cellular respiration were added. A range of concentrations of each compound was first tested until the optimal conditions were set: 8–24 µg mL^−1^ oligomycin, which blocks ATP synthase; 0.8 × 10^−6^–24 × 10^−6^ m of the uncoupler FCCP; and a mix of rotenone (3 × 10^−6^–15 × 10^−6^ m) and antimycin A (3 × 10^−6^–12 × 10^−6^ m) as inhibitors of complexes I and III in the electron transport chain, respectively. To assess adrenergic stimulation, norepinephrine was also titrated using concentrations ranging from 5 × 10^−6^ to 20 × 10^−6^ m.

The protocol is summarized in Table 1. Before the measurement, the microplate containing the tissues in AM was incubated at 37 °C without CO_2_ for 45 min. Then, the following final concentrations of the inhibitors were added after a set of titration experiments: 24 µg mL^−1^ oligomycin, 0.8 × 10^−6^ m FCCP, 5 × 10^−6^ m rotenone, and 15 × 10^−6^ m antimycin A. All the dilutions were freshly prepared. The concentrations of the inhibitors added to each corresponding port in the microplate were ten times higher, to achieve the indicated final concentrations in the assay. Each OCR measurement consisted of 3 min of mixing, 2 min wait time, and 3 min of continuous measuring of O_2_ levels. OCR was calculated by plotting the O_2_ tension of the media as a function of time (pmol min^−1^).

*Calculations*: Raw data output was transformed to OCR using the algorithm proposed by Gerencser et al.^33^ Tissue bioenergetic parameters were calculated as follows. Basal OCR was normalized by subtracting the minimum rate after rotenone and antimycin A addition (which will be referred to as nonmitochondrial OCR). ATP‐linked oxygen consumption was calculated as the difference between basal oxygen consumption and OCR, measured after the addition of oligomycin. Oligomycin resistant respiration (non‐ATP‐linked oxygen consumption or H^+^ leak) was calculated as the difference between the OCR obtained after the addition of oligomycin, minus nonmitochondrial OCR. Maximal OCR was determined by subtracting the nonmitochondrial OCR minimum reading from the maximal reading, following the addition of FCCP. Finally, the reserve capacity was calculated by subtracting the basal oxygen consumption rate from the maximal oxygen consumption after the addition of FCCP.

*Statistical Analysis*: Data were expressed as the mean ± SEM. Differences between the respiratory parameters compared to the basal OCR were analyzed using one‐way analysis of variance, followed by posthoc Tukey's multiple comparison tests. eWAT and iBAT basal OCR were compared using the Student's *t*‐test. *p* < 0.05 was considered statistically significant. All figures and statistical analyses were generated using GraphPad Prism 6 software.
